# Supplementary figures and images for: The membrane domains of mammalian adenylyl cyclases are lipid receptors
Source: eLife. 2024 Nov 29;13:RP101483. doi: 10.7554/eLife.101483 (PMC11606603; doi:10.7554/eLife.101483)

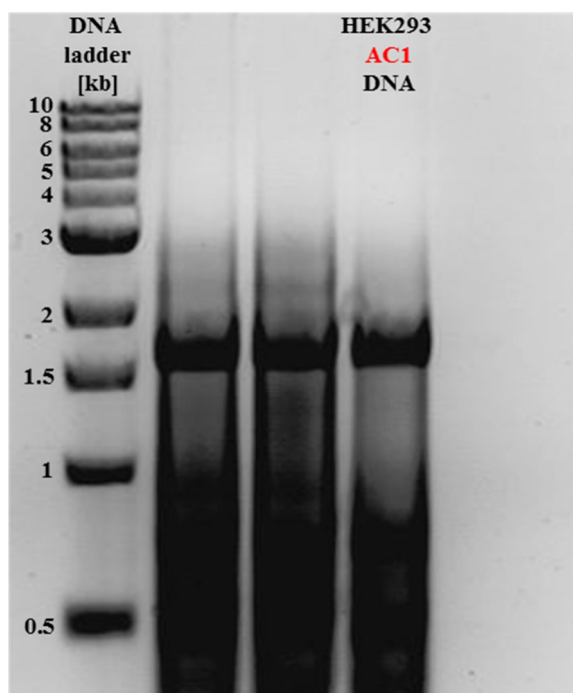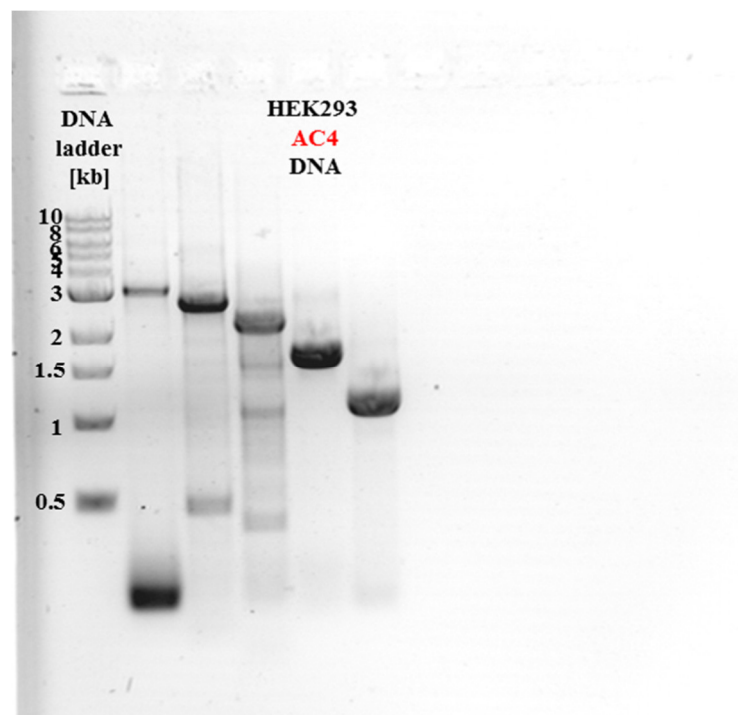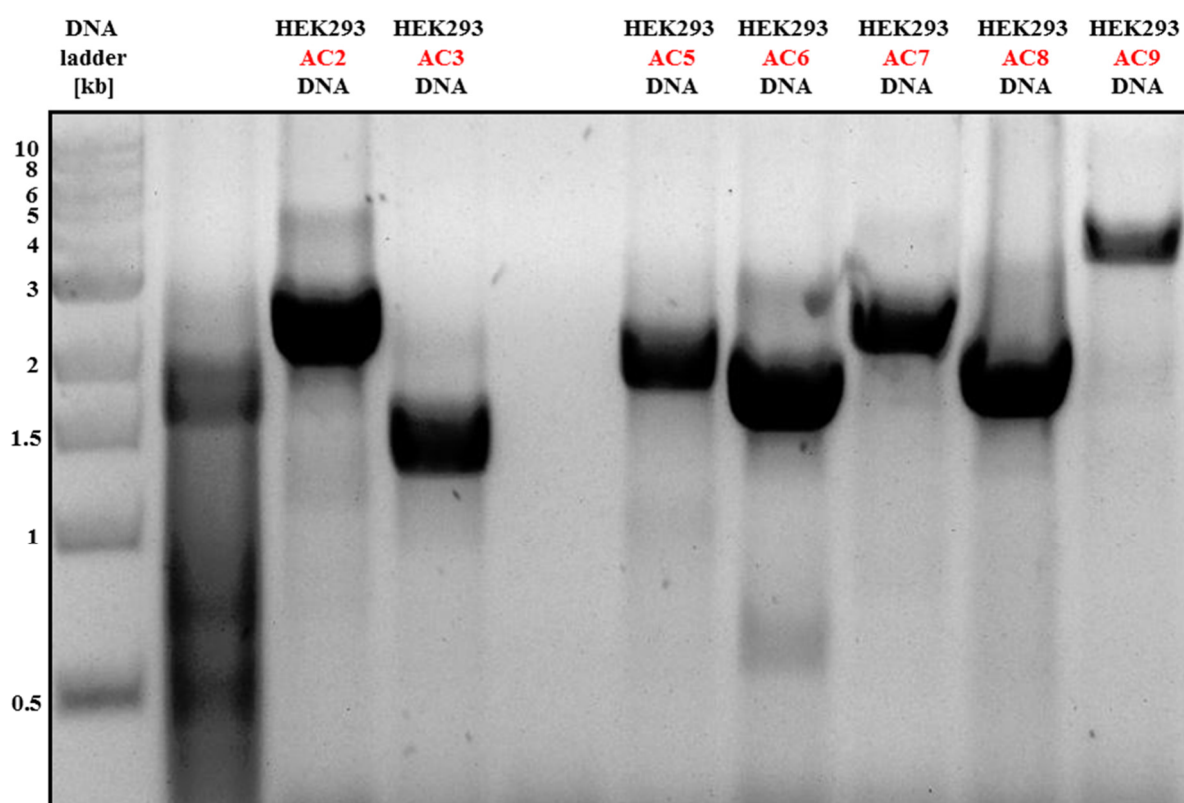

Supplement: Figure 4—figure supplement 2—source data 1. [file elife-101483-fig4-figsupp2-data1.zip › Figure 4-figure supplement 2-source data 1.pdf]

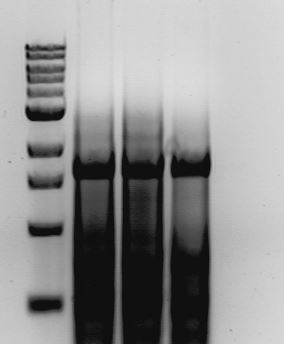

Supplement: Figure 4—figure supplement 2—source data 2. [file elife-101483-fig4-figsupp2-data2.zip › mAC1.tif]

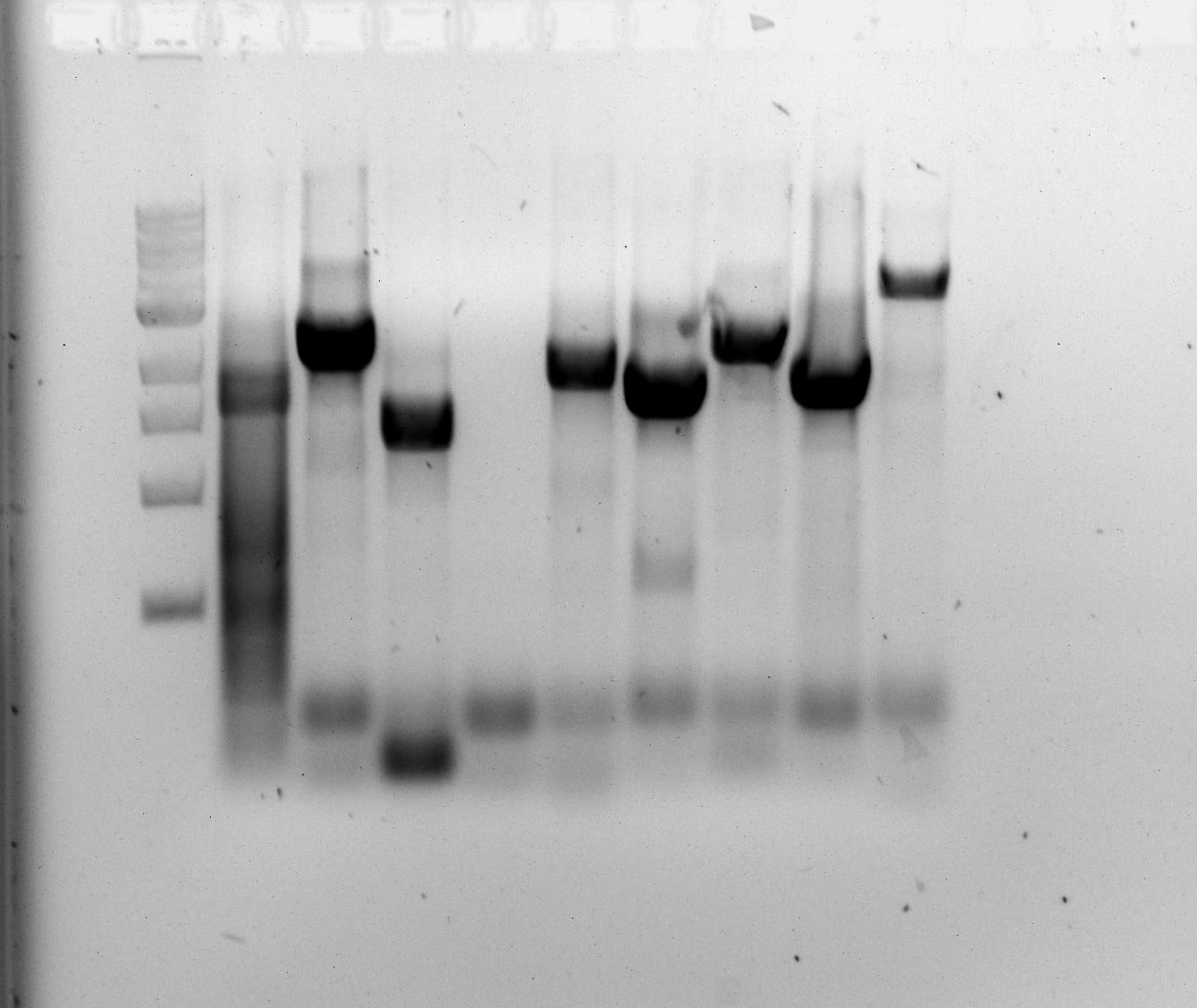

Supplement: Figure 4—figure supplement 2—source data 2. [file elife-101483-fig4-figsupp2-data2.zip › mAC2,3,5-9.tif]

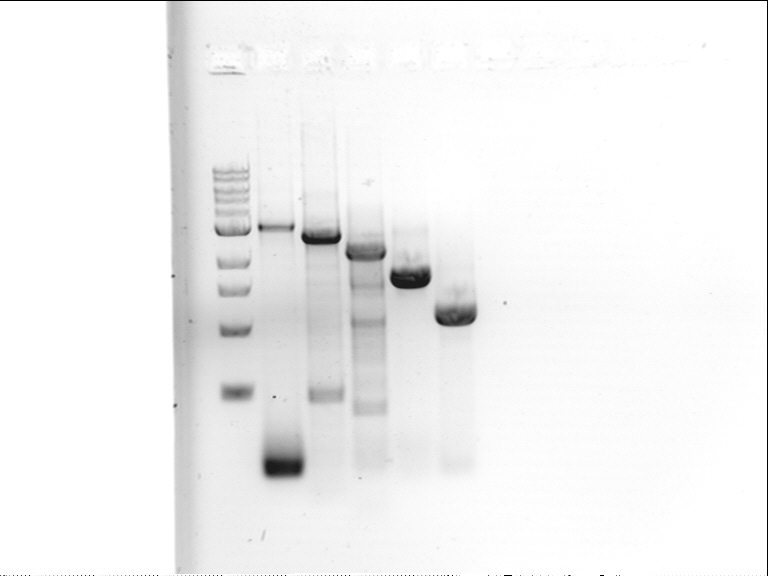

Supplement: Figure 4—figure supplement 2—source data 2. [file elife-101483-fig4-figsupp2-data2.zip › mAC4.tif]

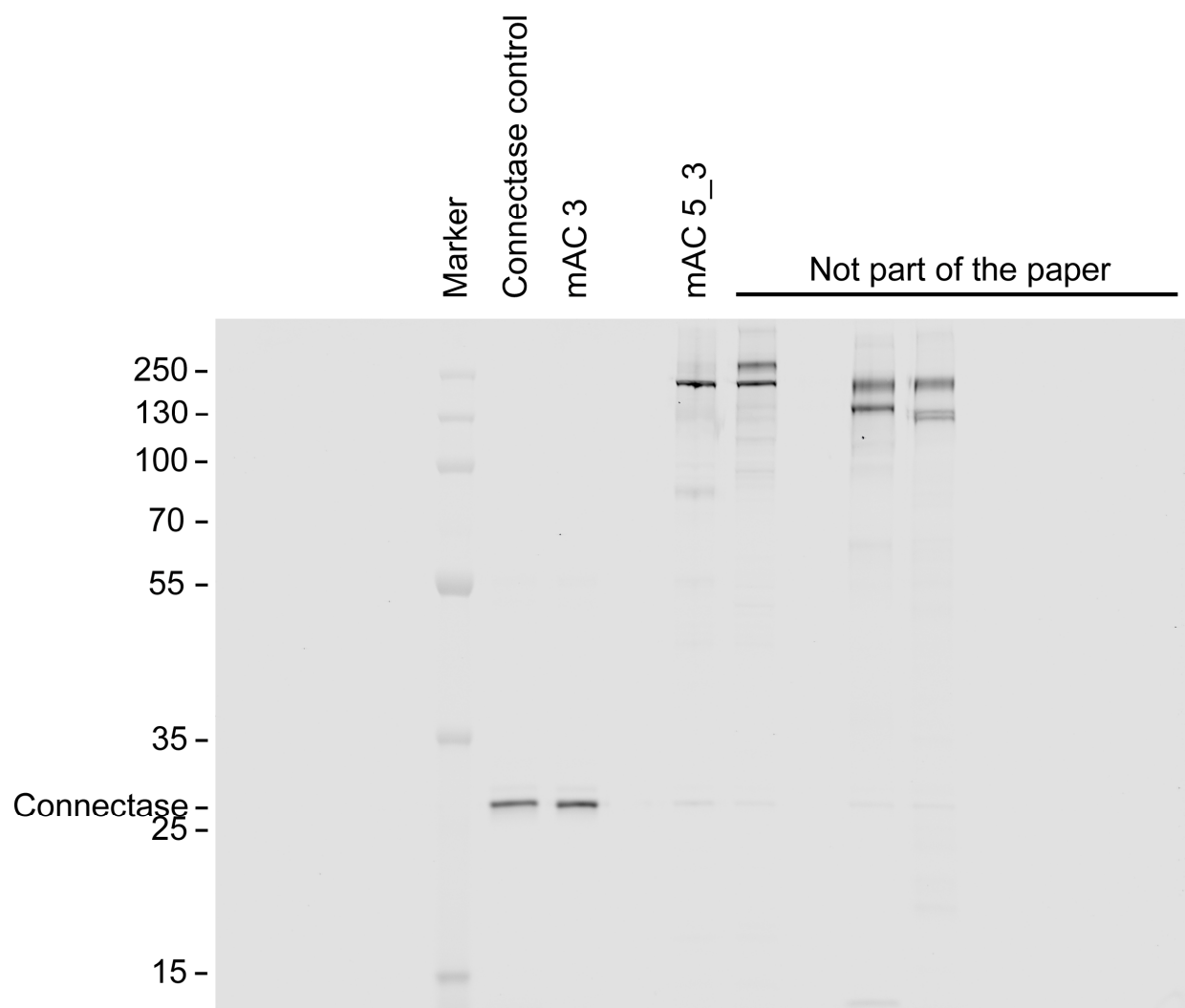

Supplement: Figure 9—source data 1. [file elife-101483-fig9-data1.zip › Figure 9 A - source data 1.pdf]
